# Deep Eutectic Solvent Based Reversed-Phase Dispersive Liquid–Liquid Microextraction and High-Performance Liquid Chromatography for the Determination of Free Tryptophan in Cold-Pressed Oils

**DOI:** 10.3390/molecules28052395

**Published:** 2023-03-05

**Authors:** Slavica Ražić, Tamara Bakić, Aleksandra Topić, Jelena Lukić, Antonije Onjia

**Affiliations:** 1 Faculty of Pharmacy, University of Belgrade, Vojvode Stepe 450, 11221 Belgrade, Serbia; 2 Innovation Center of the Faculty of Technology and Metallurgy, Karnegijeva 4, 11120 Belgrade, Serbia; 3 Faculty of Technology and Metallurgy, University of Belgrade, Karnegijeva 4, 11120 Belgrade, Serbia

**Keywords:** RP-DLLME, nuts, seeds, ionic liquids, factorial design, chemometric optimization, Plackett–Burman, HPLC

## Abstract

A fast and straightforward reversed-phase dispersive liquid–liquid microextraction (RP-DLLME) using a deep eutectic solvent (DES) procedure to determine free tryptophan in vegetable oils was developed. The influence of eight variables affecting the RP-DLLME efficiency has been studied by a multivariate approach. A Plackett–Burman design for screening the most influential variables followed by a central composite response surface methodology led to an optimum RP-DLLME setup for a 1 g oil sample: 9 mL hexane as the diluting solvent, vortex extraction with 0.45 mL of DES (choline chloride–urea) at 40 °C, without addition of salt, and centrifugation at 6000 rpm for 4.0 min. The reconstituted extract was directly injected into a high-performance liquid chromatography (HPLC) system working in the diode array mode. At the studied concentration levels, the obtained method detection limits (MDL) was 11 mg/kg, linearity in matrix-matched standards was R^2^ ≥ 0.997, relative standard deviations (RSD) was 7.8%, and average recovery was 93%. The combined use of the recently developed DES -based RP-DLLME and HPLC provides an innovative, efficient, cost-effective, and more sustainable method for the extraction and quantification of free tryptophan in oily food matrices. The method was employed to analyze cold-pressed oils from nine vegetables (Brazil nut, almond, cashew, hazelnut, peanut, pumpkin, sesame, sunflower, and walnut) for the first time. The results showed that free tryptophan was present in the range of 11–38 mg/100 g. This article is important for its contributions to the field of food analysis, and for its development of a new and efficient method for the determination of free tryptophan in complex matrices, which has the potential to be applied to other analytes and sample types.

## 1. Introduction

Tryptophan (Trp), an essential amino acid, is a precursor of many biologically active substances, including serotonin, melatonin, quinolinic acid, kynurenic acid, and tryptamine, as well as coenzymes important for electron transfer reactions (redox balance of metabolism), such as nicotinamide adenine dinucleotide (NAD+) [1]. A variety of pathological processes in humans are caused by the disorders of tryptophan metabolism, including neurologic disorders, inflammatory bowel disease, malignancies, and cardiovascular disease [2].

The human body cannot synthesize Trp and is dependent on dietary sources of Trp. It is found in foods that naturally contain protein, in dietetic and fortified food products, and in specific pharmaceutical formulations [3]. Nuts and seed oils are particularly rich in Trp [4,5]. Thus, detecting and quantifying this compound in vegetables is needed, but it is a significant analytical challenge [1].

Many analytical techniques have been recently investigated for Trp detection and quantification, including colorimetry [6,7,8,9], fluorimetry [10], voltammetry [11,12,13,14,15], capillary electrophoresis [16,17], gas chromatography–mass spectrometry (GC-MS) [18], high-performance liquid chromatography (HPLC) [19,20,21,22,23,24,25,26,27,28], and HPLC single [29] or tandem mass spectrometry (HPLC-MS/MS) [30,31,32,33,34,35]. Among these techniques, HPLC has been the most widely used due to its high selectivity and good sensitivity for quantifying Trp.

Since the complex matrix of food samples, which often contain large quantities of interfering substances, may influence the Trp content determination [36], a sample pre-treatment is necessary. Acid hydrolysis is used in the analytical protocols of most amino acids [36]. However, when determining protein-bound Trp in food samples, it was shown that good results could only be obtained after the preparation of samples by alkaline hydrolysis [23] or enzymatic hydrolysis [25]. In any case, an additional extraction step prior to instrumental measurements is unavoidable. Whereas protein-bound Trp can be determined if only a sample hydrolysis step is included in the sample preparation procedure, the hydrolysis may be omitted if the content of free Trp is determined [37].

Time-consuming solid-phase extraction (SPE) and conventional liquid–liquid extraction (LLE), which need a large quantity of potentially toxic solvents [38,39], are recently being replaced with greener, miniaturized sample preparation techniques [40,41,42]. Numerous methods have been developed for this purpose, of which dispersive liquid-liquid microextraction (DLLME) has proven to be effective, simple, fast, economical, and environmentally friendly [43,44,45,46].

Reversed-phase DLLME (RP-DLLME) is a new modification of DLLME in which an extraction solvent compatible with the HPLC mobile phase is used [47,48]. In RP-DLLME, a small volume of extraction solvent is rapidly dispersed in the hydrophobic sample solution using a dispersive solvent to form a fine droplet phase. The analytes partition into the aqueous extraction phase, which is then collected and analyzed. The aqueous phase can often be injected directly into an HPLC system. In this way, the time required for solvent evaporation is saved. RP-DLLME has recently been used for the enrichment and extraction of a wide range of analytes from various lipophilic sample matrices, including biological samples [49], cosmetics [50], and vegetable oils [51,52,53].

The recently introduced DLLME using a deep eutectic solvent (DES) has attracted tremendous attention [54,55,56,57,58]. DES represents a mixture of a hydrogen bond donor (HBD) and hydrogen bond acceptor (HBA). Owing to the hydrogen bond interactions, this mixture is able to self-associate and form a eutectic mixture with a lower melting point than those of the individual constituents [57]. Unlike traditional ionic liquids with similar extraction properties, these compounds are more environmentally friendly and cheaper. In addition, hydrophilic DESs have the advantage of being compatible with the RP-HPLC mobile phase [59].

This work investigates a new analytical method for separating and determining free Trp in an oily matrix using DES–RP-DLLME followed by an HPLC–diode array detector (DAD). The DES–RP-DLLME variables were optimized using the design of experiments (DoE) [60,61]. The method was validated and applied to determine free Trp in cold-pressed vegetable oils.

The novelty of this study lies in the development of a method for the determination of free Trp in cold-pressed oils using a combination of DoE-optimized DES–RP-DLLME and HPLC. It offers several advantages over conventional extraction techniques, including improved selectivity and reduced solvent consumption. A small volume of DES, used as the extraction solvent, provides additional benefits, including its low toxicity, biodegradability, and cost effectiveness, making it an environmentally friendly and efficient choice for sample preparation.

## 2. Results and Discussion

### 2.1. Selection of DESs

Seven hydrophilic DESs based on ChCl receptor bonds have been evaluated as candidates for the RP-DLLME extractant. Two DESs, ChCl:DA and ChCl:CA, were not clear liquids at room temperature.

**Figure 1:**
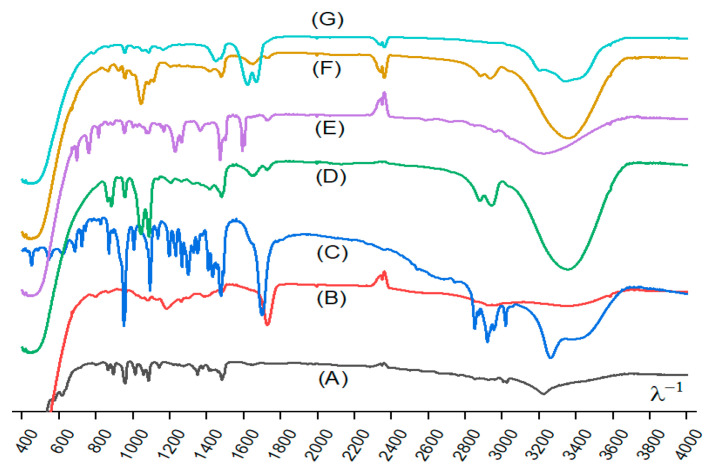
FTIR spectra of the synthesized deep eutectic solvents. (**A**) ChCl:W; (**B**) ChCl:CA; (**C**) ChCl:DA; (**D**) ChCl:EG; (**E**) ChCl:Ph; (**F**) ChCl:G; and (**G**) ChCl:U.

FTIR spectra of these DESs placed in pressed KBr tablets are illustrated in Figure 1. A comparison between the spectrums ChCl and its DES mixtures was made in order to identify the changes in the structure and the new interactions between the constituents in the synthesized DESs. It can be noted that ChCl has retained its structure, as some of its peaks were also observed in the DES spectrums. The presence of water had an insignificant effect on the vibration frequencies in the formed ChCl:water DES [62]. The bands related to pure ChCl compared to those related to the DESs showed a small frequency deviation and change in bandwidth [63]. Thus, the positions of some characteristic ChCl peaks, symmetrical C-H stretch at 2800 cm^−1^, and asymmetric C-CH_3_ stretch at 3000 cm^−1^ were changed in the DESs

The OH vibration of the ChCl at 3200 cm^−1^ was shifted to 3400 cm^−1^. This shift and the broadening of the O-H vibration bands indicate the presence of hydrogen bonds between ChCl and donor compounds when the DES is formed. This may be attributed to the transfer of oxygen atom cloud electrons to hydrogen bonding [64]. The peak at 1000 cm^−1^ is indicative of the C-N vibration. The carboxylic group at 1700 cm^−1^ can be observed in ChCl:CA and ChCl:DA. In the spectra of ChCl:DA, a polyunsaturated fatty acid chain is represented by the band at 3000 cm^−1^ [65,66], while the frequencies of 1600 cm^−1^ indicate the vibration of N-H and C-N in ChCl:U. At 1100 cm^−1^, C-N bond vibration is shown.

These DESs were tested as the candidate solvent for RP-DLLME of Trp from oils. For this purpose, all experimental variables were set to the middle point in the RP-DLLME experimental domain (Table 1). Enrichment factor (EF), calculated as the ratio between the Trp concentration in the final DES solution (C_f_) and the Trp concentration in the initial oil sample (C_i_), was used to estimate the extraction recovery (ER) applying the following equation:

*ER* = 100 × *EF* × (*V_f_*/*V_i_*) (1)

where *V_i_* and *V_f_* are the initial sample volume and the volume of the final reconstituted DES extract, respectively.

**Figure 2:**
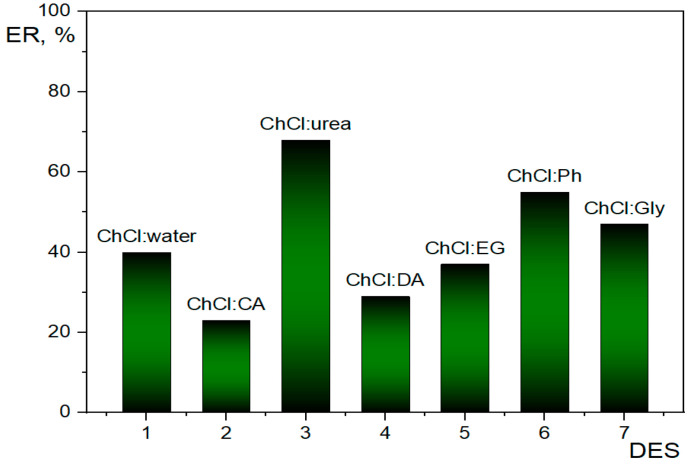
Extraction efficiency of different deep eutectic solvents for RP-DLLME of tryptophan from oil.

Figure 2 shows the comparison of ERs in RP-DLLME of Trp from spiked samples for seven DESs. It is obvious that ChCl:U is capable of extracting the highest amount of Trp from an oily matrix (68%). In addition, this DES gives a clear solution and is straightforward for RP-DLME. In general, the results revealed that ChCl:U was the most suitable DES compared to other ones.

ChCl:urea was able to extract free Trp from an oily matrix by a combination of several intermolecular forces [50]. One of the most important forces is the hydrogen bonding that occurs between the hydrogen atom of the hydroxyl group of choline chloride and the nitrogen and oxygen atoms of the functional groups of Trp. This interaction facilitates the transfer of Trp from the sample matrix to the Chl:urea phase. Another important force is the hydrophobic interaction that occurs between the hydrophobic alkyl chains of the ChCl:urea DES and the nonpolar side chains of Trp. This interaction promotes the partitioning of Trp from the sample matrix into the ChCl:urea phase because Trp prefers to interact with nonpolar molecules rather than polar molecules. Furthermore, some additional intermolecular forces, such as electrostatic and van der Waals forces, contribute to the extraction mechanism. Unlike other DESs, both the ChCl and urea components of the DES have the ability to form hydrogen bonds, which increases the solubilizing power for Trp. Indeed, Trp is an aromatic amino acid with polar and nonpolar moieties, which has a relatively high polarity due to the presence of amino and carboxyl groups and can form hydrogen bonds with the polar functional groups of ChCl and urea.

### 2.2. Optimization of DLLME Procedure

Optimal microextraction of Trp was obtained by optimizing eight RP-DLLME variables in two steps. At first, the data obtained from the PBD experiments were analyzed using the ANOVA test, and the results are shown in Table 2. This helped in precisely choosing the variables with the largest influence using a minimal number of experiments. Here, the temperature and the DES amount appeared to be the two most influential variables, with the highest F-values (30.8 and 12.1). It was also observed that increasing the diluting factor, the extraction time, the centrifugation rate, and decreasing the amount of added salt and the centrifugation time resulted in an increase in ER. At the same time, vortexing was found to give better results than sonicating.

**Figure 3:**
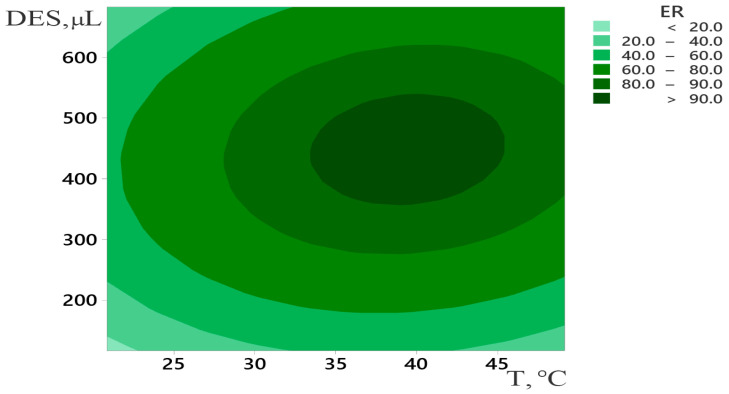
Response surface plot for optimization of RP-DLLME of tryptophan.

The next step in the optimization used RSM to find the best ER from RP-DLLM of Trp. In this case, the experimental domain was extended to 700 μL of DES. Figure 3 presents the response surface plot of ER as a function of the temperature and the DES amount. This method included quadratic and interaction terms in the model. Therefore, it was possible to account for the detailed effects of selected variables on each other and also on ER.

Thus, the experimental data were fitted by a second-order polynomial model (Equation (2)), which consisted of two main effect terms, two two-factor interaction effect terms and two curvature effect terms. The regression equation is

*ER* = *A* + *B*∙*T* + *C*∙*DES* + *D*∙*T*^2^ + *E*∙*DES*^2^ + *F*∙*T*∙*DES* (2)

where *A* (−144.2), *B* (7.92), *C* (0.3656), *D* (0.1087), *E* (−0.000472), and *F* (0.00147) terms were optimized iteratively to fit the model.

Finally, the optimized RP-DLLME variables may be summarized as follows: initial sample dilution ratio of 1:9, DES amount 450 μL, extraction time 3 min, extraction temperature 40 °C, no salt addition, vortex stirring type, centrifuge speed 6000 rpm, and centrifugation time 4 min.

Because samples containing a significant amount of tryptophan were analyzed in this study, an enrichment factor of 2.2 was sufficient for quantitative analysis.

### 2.3. Validation of RP-DLLME-HPLC Method

**Figure 4:**
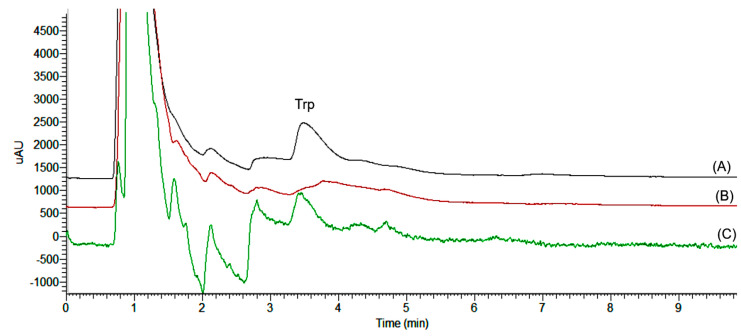
HPLC chromatograms of free tryptophan after RP−DLLME. (A) Matrix-matched standard (20 mg/kg 100 g oil); (B) blank; and (C) almond oil sample.

Analytical figures of the current method were determined using spiked samples. A typical chromatogram obtained under the optimum DES-RP–DLLME–HPLC–DAD conditions for Trp in a cold-pressed almond oil sample is shown in Figure 4.

The method was validated by referring to US FDA official guidance [67]. The performance of the analytical method was evaluated by considering the recovery, precision, limit of detection (LOD), and matrix effect.

Six different calibration standards in the range of 10–400 mg/kg of Trp in methanol were analyzed by HPLC–DAD to obtain the linearity of the method. In addition, the matrix-matched standards at the same concentration levels were measured after RP-DLLME. The matrix effect (ME) was estimated by using the ratio between the slope of the calibration curve of the standards in methanol (b_1_ = 923,541) and the slope of the matrix-matched calibration curve (b_2_ = 775,774). In both cases, the linear correlation coefficients were r^2^ > 0.996. However, the estimated ME was 26%, which indicates a matrix-induced suppression of the analytical signal.

According to the FDA guideline, limit of detection (LOD) values should be ≥ten times lower than quantified concentrations in the 0.1–10,000 mg/kg range. The LOD value of Trp was selected to be the concentration that gave the signal-to-noise ratio (S/N = 3) for the Trp peak which was 11 mg/kg. This LOD value allows the determination of Trp in cold-pressed vegetable oils.

Precision was evaluated by determining the RSD of six replicate spiked samples at three concentrations (50, 200, and 400 mg/kg), and it was determined to be 9%, 6%, and 5%, respectively. The predicted relative reproducibility standard deviation acceptable by the FDA for the concentration levels of 10, 100, and 1000 mg/kg are 11, 8, and 6%, respectively.

The average recovery for the same spikes was found to be 91%, 94%, and 93%, respectively. The FDA required recoveries for a quantitative method at the concentration level of 100 mg/L to be 90–107%.

### 2.4. Critical Analysis of the Method Performances

Although hydrophilic ChCl-based DESs have recently been used as an extraction solvent for RP-DLLME [58,67,68,69], no published method has been found for DLLME of Trp from foods The proposed method for the determination of Trp in vegetable oils using DoE-optimized DES–RP-DLLME and HPLC has a unique combination of features that make it a highly efficient and environmentally friendly technique. Comparing the developed RP-DLLME method for free Trp determination in oil samples with other reported methodologies reveals its pros and cons (Table 3). Electroanalytical techniques such as voltammetry cannot compete with chromatographic techniques in terms of separation power. For the analysis of real samples of complex matrices, only HPLC and LC-MS can be used. The latter has a much higher sensitivity. However, the detector MS is complex and expensive, requires extensive sample preparation, and is often not available in a small laboratory. It is likely that the current HPLC method requires a simpler and less expensive procedure that increases labor while reducing analytical costs.

Unlike simple extraction or dilution in the analysis of hydrophilic samples, an oily matrix requires greater removal of interferences. An alternative technique to RP-DLLME, which can achieve lower LODs, is solid phase extraction (SPE). However, SPE consumes more organic solvents and requires an SPE manifold. Considering that sample cleanup is required for nuts and seed oils and the tryptophan concentration in the samples tested is high, the sensitivity of this method is quite acceptable.

In general, the green analytical chemistry (GAC) aspects of this method are its main advantages. All 12 GAC principles affecting the quality attributes of the analytical method were addressed here: green (G1. Toxicity of reagents, G2. Number and amount of reagents and waste, G3. Energy, G4. Direct impacts on the human), red (R1. Scope of application, R2. LOD and LOQ, R3. Precision, R4. Accuracy), and blue (B1. Cost-efficiency, B2. Time-efficiency, B3. Minimal requirements, B4. Operational simplicity).

Three greenness assessment approaches [70,71,72] were used to evaluate the environmental impact of this method: Analytical Eco-Scale, Green Analytical Procedure Index (GAPI), and Analytical Greenness metric (AGREE).

The Analytical Eco-Scale tool is used to assess the greenness level of analytical procedures in terms of the number of hazards. This method assigns penalty points for the amount and type of reagents used, hazards, energy consumed, and waste generated. The penalty points are then subtracted from a value of 100. It is considered an excellent green method if the method receives a score of ≥75. The method is considered inadequate if the Eco-scale value is less than 50. A high Eco-Scale score is primarily attributed to the amount and type of solvents consumed. The calculated penalty points (14) for our method resulted in an Eco-scale score of 86 out of 100, indicating that the developed method was excellent green (Supplementary Table S1).

**Figure 5:**
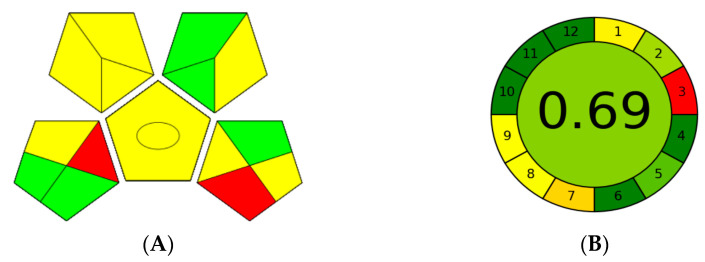
Greenness assessment of the DES–RP-DLLME-HPLC method for tryptophan in oils. (**A**) GAPI pentagram; (**B**) AGREE pictogram.

GAPI evaluates the greenness level of an analytical method through five fields. Each field represents a different aspect of the developed method (Supplementary Table S2). Fields are colored green, yellow, or red depending on the ecological impact of each step. In this work, the GAPI pentagram had five green, seven yellow, and two red fields (Figure 5A). One of the red fields is because this method is not an in-, at-, or on-line method. Another pentagram is shaded red because sample preparation is required.

The AGREE method assesses the environmental impact of the method using a pictogram divided into twelve sections, which correspond to the twelve GAC principles. Each section and the middle zone of the AGREE pictogram is colored from green to red. According to the method greenness, the total score of the method is calculated and appears in the middle zone of the AGREE pictogram. The AGREE score in this study was 0.69 (Figure 5B), indicating that the method is environmentally friendly and has no negative impact on the environment.

### 2.5. Analysis of Real Samples

Five samples of each vegetable were purchased in different stories and analyzed (Supplementary Table S3). The analytical results of free Trp in different vegetable oils, using the DES–RP-DLLME–HPLC–DAD method, are presented in Table 4. The results, expressed in mg of Trp per 100 g of cold-pressed oils, are in the range between 11 and 38 mg/100 g oil.

Sunflower seed oil shows the highest average Trp concentration (38 mg/100 g), while cashew oil has the lowest (11 mg/100 g). Trp levels significantly differed among the nut and seed samples. In general, cold-pressed oils from seeds have higher Trp content than those from nuts.

## 3. Materials and Methods

### 3.1. Reagents and Chemicals

L-Tryptophan, reagent grade standard (≥98% purity), was purchased from Sigma-Aldrich Chemie GmbH (Taufkirchen, Germany). The stock solution (100 mg/kg) of Trp was prepared in methanol/deionized water (50:50 *v*/*v*). The working solutions were prepared by appropriately diluting the stock solution with methanol/deionized water (50:50 *v*/*v*). Methanol, acetonitrile (HPLC-grade), and sodium chloride (99%) were purchased from Merck KGaA, (Darmstadt, Germany). All standard solutions were stored at 4 °C and brought to an ambient temperature just prior to use.

Seven different types of DESs, at a molar ratio of 1:2, were prepared by mixing choline chloride:urea (ChCl:U), choline chloride:phenol (ChCl:Ph), choline chloride:citric acid (ChCl:CA), choline chloride:decanoic acid (ChCl:DA), choline chloride:glycerol (ChCl:G), choline chloride:ethylene glycol (ChCl:EG), and choline chloride:water (ChCl:W). The mixtures were stirred at 80 °C until DESs were formed. These DESs were used for RP-DLLME of Trp from vegetable oil samples.

### 3.2. Sample Preparation

**Figure 6:**
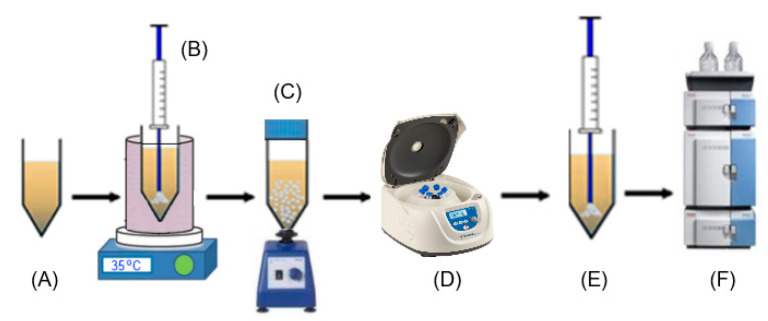
Scheme of the RP-DLLME-HPLC procedure. (**A**) Diluted oil sample; (**B**) injection of DES; (**C**) vortexing; (**D**) centrifugation; (**E**) retrieval of the DES phase; (**F**) HPLC measurement.

Nine vegetable samples (sunflower seeds, sesame seeds, peanuts, cashews, Brazil nuts, pumpkin seeds, almonds, walnuts, and hazelnuts) underwent cold pressing using a press model VitaWAY OP650W (Gorenje d.o.o. Valjevo, Serbia), and the produced oils were stored at 4 °C until analysis. Trp was extracted from the oils using the following RP-DLLME procedure (Figure 6).

First, a 1.0 g sample measured in a 15 mL amber glass centrifuge tube was diluted to an appropriate ratio by adding n-hexane. Next, a volume of a DES, as the extractant, was rapidly added to the sample solution. The resulting mixture was vigorously shaken using a vortex agitator or ultrasonic bath. After centrifugation, two clear phases were observed, and the DES phase containing the extracted Trp was settled at the bottom of the tube. Next, an aliquot of the DES phase (lower phase) was withdrawn through a syringe and diluted with methanol. Finally, 5 µL of this solution was injected into an HPLC–DAD system for measurement. Spiked samples were prepared by adding an appropriate amount of Trp into a linoleic acid/oleic acid (1:1) mixture and processed in the same way.

A vortex (IKA model MS2 Mixer, IKA-Werke GmbH, Staufen, Germany) or ultrasonic bath (model Eumax 3L 100W, Skymen Cleaning Equipment Co. Ltd., Shenzhen, China) was used for mixing, whereas the phase separation was performed using a centrifuge (model Colo Lace16AS, Colo d.o.o., Rogatska Slatina, Slovenia). The extraction was performed in a temperature-controlled, shaking water bath (Memmert, model WNB 14, Memmert GmbH, Schwabach, Germany). Fourier-transform infrared (FTIR) spectra of the DESs were recorded in the wavelength range from 500 to 4000 cm^−1^ on a Thermo model Nicolet iS10 FTIR spectrometer equipped with the Omnic software (Thermo Electron Scientific Instruments LLC, Madison, WI, USA).

### 3.3. RP-DLLME Optimization

The RP-DLLME procedure has been optimized using the Plackett–Burman screening design (PBD) [46] for variable selection, followed by the central composite design (CCD) [45] used to find the optimal values for the variables. Table 1 shows the eight investigated RP-DLLME variables and their ranges.

After PBD screening experiments, thew variables that significantly influenced the RP-DLLME process were optimized following a response surface methodology using CCD.

### 3.4. HPLC Measurements

The Trp quantification was performed using an HPLC system comprising a pump, autosampler, and diode array detector (model Accela, Thermo Fisher Scientific Inc., Waltham, MA, USA). Isocratic elution at a 1.0 mL/min flow rate was used on a Thermo Scientific Hypersil ODS (C18) Column (5 μm particle size, length 100 mm, 4.6 mm I.D.) at 35 °C. The mobile phase consisted of methanol:water (with 1% acetic acid) (40:60 *v*/*v*). The DAD spectrum was continuously recorded along with UV detection at a wavelength of 280 nm. The retention time for Trp was 3.54 min.

### 3.5. Method Validation

Analytical method validation was conducted using a mixture of linoleic:oleic (1:1) acids spiked with Trp at different levels. Linearity and the matrix effect were determined using calibration standards in methanol and matrix-matched standards. Recovery and relative standard deviation (RSD) values were determined using the spiked replicate samples.

### 3.6. Software

Statistical analysis for Plackett–Burman (PBD) design and ANOVA test was conducted using a software package of Minitab (release ver. 13.20).

## 4. Conclusions

RP-DLLME combined with HPLC–DAD was developed to determine the free Trp content of cold-pressed nuts and seed oils. A DES solvent comprised of choline chloride and urea was found as the most suitable. Plackett–Burman screening DoE, followed by the central composite RSM, was employed to estimate the optimum RP-DLLME conditions that yield the maximum extraction efficiency. Good analytical recovery and RSD for the method were obtained by analyzing spiked replicates. Applying this method, free Trp was determined in cold-pressed oils from cashews, walnuts, Brazil nuts, almonds, hazelnuts, peanuts, pumpkin seeds, sesame seeds, and sunflower seeds at the levels of 11, 12, 14, 16, 17, 18, 32, 33, and 38 mg/100 g, respectively. The findings of this study suggest that the use of DES-based methods for extraction and analysis of target analytes in complex food matrices can contribute to sustainable development in the analytical chemistry and food industry and may serve as a model for the development of greener and more sustainable food analytical methods in the future.

## Figures and Tables

**Table 1:** Variables and their ranges (−1, 0, +1) for the Plackett–Burman screening design.

No.	Variable	Symbol	Level		
			−1	0	+1
1.	Initial sample dilution ratio (:)	dil	1:1	1:5	1:9
2.	DES amount (μL)	DES	100	200	300
3.	Extraction time (min)	t_ex_	1	3	5
4.	Extraction temperature (°C)	T	25	35	45
5.	Salt (NaCl) addition (%)	salt	0	5	10
6.	Stirring type (vortex or ultrasonic)	stir	Vor	-	Us
7.	Centrifuge speed (rpm)	w	2000	6000	10,000
8.	Centrifuge time (min)	t_cfg_	2	6	10

**Table 2:** ANOVA results from the Plackett–Burman (PBD) design.

Source	DF	Adj SS	Adj MS	F-Value	*p*-Value
Model	8	2222	277.7	8.97	0.049
Linear	8	2222	277.7	8.97	0.049
dil	1	184.0	184.1	5.94	0.093
DES	1	374.0	374.1	12.1	0.040
t_ex_	1	24.08	24.08	0.78	0.443
T	1	954.0	954.0	30.8	0.012
salt	1	270.7	270.7	8.78	0.060
stir	1	310.1	310.1	10.01	0.051
W	1	90.75	90.75	2.93	0.185
t_cfg_	1	14.08	14.08	0.45	0.548
Error	3	92.92	30.97		
Total	11	2314			

**Table 3:** Comparison of the proposed method with other reported methods for the determination of tryptophan.

No.	Matrix	Concentration Range	Sample Preparation Method	Reagents/ Extractant	Analytical Technique	Limit of Detection	Linearity (R^2^)	Recovery (%)	RSD (%)	Reference
1	Protein	10-100 μg	acid hydrolysis	HCl/ninhydrin	ViS	n.a.	n.a.	98.3	2.7	[6]
2	Yeast extract *	100–600 μM	enzymatic hydrolysis	hydroxylamine	ViS	100 μM	0.6969	86	n.a.	[7]
3	Solution *	10–100 mg/L	oxidation	NaOCl	ViS	10 mg/L	0.9996	90.5	1.19	[8]
4	Millets *	9–36 mg/L	biorecognition	MP@PDA-*E. coli*	ViS	5.6 μM	0.98	106	7.3	[9]
5	Beer *	0.02–0.12 mg/L	dSPE	graphene/clay/ Brij L23	FL	0.01 mg/L	0.9991	90	5.0	[10]
6	Dietary supplements *	1.0–7.0 μmol/L	dilution	GCE/p-ARG	SWV	0.30 μmol/L	0.990	97.6	2.1	[11]
7	Plasma *	0.08–20.0 μM	screen-printed electrode	PdCuCo/RGO	DPV	0.03 μM	0.997	103.7	2.8	[12]
8	Milk *	5.0–150 µM	electrochemical sensor	graphite electrode	DPV	5.78 µM	0.9841	99.3	8.6	[13]
9	Pharmaceutics *	1–350 μM	MIP electrochemical sensor	AuNPs@PVP@SiO2MIP	LSV	1 μM	0.995	105	4	[14]
10	Milk *	0.01–80 μM	MIP biosensor	MIP-AF	EIS	0.008 μM	0.99	98.2	1.8	[15]
11	Plasma *	0.005–0.1 mol/L	dilution	HBP/SSA	CE	5 μmol/L	0.998	101.9	5.4	[16]
12	Beer *	n.d.–40.7 mg/L	acid hydrolysis	HCl/HEC//BTP/EACA/AMPD	cITP	4.35 mg/L	0.9993	95.9	4.3	[17]
13	Leaf tissue *	n.a.	SPE	acetic anhydride deriv.	GC-MS	n.a.	n.a.	60	2	[18]
14	Soy sauces *	136–262 mg/L	precipitation	ethanol	HPLC	1 mg/L	0.995	108	4.9	[19]
15	Dietary supplements *	5.0–500 μg/m	HILIC	1-octane sulfonate	HPLC	1.2 mg/mL	0.979	96.5	2.3	[20]
16	Infant formula	0.018–30 mg/kg	enzymatic hydrolysis	pronase enzyme	HPLC	18 μg/kg	0.9999	93.8	6.9	[21]
17	Rapeseed	10–400 ng	alkaline hydrolysis	NaOH	HPLC	10 ng	0.998	98.6	1.6	[22]
18	Pig feed *	n.a.	dilution	HCl	HPLC	n.a.	n.a.	n.a.	5.0	[23]
19	Chicken feed	59–130 g/kg	alkaline hydrolysis	NaOH/o-phthalaldehyde	HPLC-FLD	n.a.	n.a.	86	4.0	[24]
20	White bread		alkaline hydrolysis	NaOH	HPLC	n.a.	n.a.	85	16.1	[25]
21	Wheat	1.3–14.8 g/kg	alkaline hydrolysis	NaOH/O-phthalaldehyde	HPLC-FLD	n.a.	n.a.	91.6	1.9	[26]
22	Yogurt	352–1220 mg/kg	alkaline hydrolysis	NaOH/5-methyl-l-tryptophan	HPLC-FLD	11 μg/kg	0.9995	93	1.1	[27]
23	Bee pollen *	0.069 mg/g	ultrasonic extraction	ACN	HPLC-FLD	0.003 mg/L	0.9998	93.8	3.82	[28]
24	Ryegrass shoot	0.5–40 µM	alkaline hydrolysis	NaOH	LC-MS	0.02 µM	0.99	89.9	8.5	[29]
25	Whole blood	0.1–25 ng/mL	VAMS	ACN/H_2_O	LC-MS/MS	25 ng/mL	0.9987	85	9.6	[30]
26	Plasma	0–160 μM	acid hydrolysis	MeOH/ZnSO_4_/TFA	LC-MS/MS	83 nM/L	0.995	88	11	[31]
27	Honey *	0.7–9.94 mg/kg	SPE	Oasis MCX 30 µm	LC-MS/MS	1.0 μg/kg	n.a.	60	4.3	[32]
28	Milk *	89.6–117	QuEChERS	CAN	LC-MS/MS	2 ng/mL	0.99	103.7	2.6	[33]
29	Plant material *	1–50 ng/mL	SPE	Hybrid SPE–phospholipids	LC-MS/MS	4 ng/mL	0.996	87.8	15	[34]
30	Chicken feed	n.a.	microwave hydrolysis	AQC-derivatization	LC-MS/MS	1 fmol	n.a.	99	4.2	[35]
31	Hazelnut *	42–127 μg/g	water extraction	water	UPLC-MS/MS	n.a.	n.a.	117	30	[37]
32	Nuts and seed oils *	10–400 mg/kg	RP-DLLME	DES (ChCl:U)	HPLC	11 mg/kg	0.996	91	9.0	This study

*—free Trp was analyzed; ViS—visible spectrophotometry; DPV—differential pulse voltammetry; LSV—linear sweep voltammetry; SWV—square wave voltammetry; MIP—molecularly imprinted polymer; EIS—electrochemical impedance spectroscopy; VAMS—volumetric absorptive microsampling; FL—fluorescence; FLD—fluorescence detector; CE—capillary electrophoresis; cITP—capillary isotachophoresis; dSPE—dispersive solid phase extraction; SPE—solid-phase extraction; HILIC—hydrophilic interaction liquid chromatography; n.a.—not available; n.d.—not detected.

**Table 4:** Trp (free) content in vegetable oils.

No.	Oils Made from	Trp Content (mg/100 g)	±	Variation of Trp Content between Samples (%)
1.	Almonds	16	±	16
2.	Brazil nuts	14	±	18
3.	Cashews	11	±	15
4.	Hazelnuts	17	±	14
5.	Peanuts	18	±	13
6.	Pumpkin seeds	32	±	26
7.	Sesame seeds	33	±	27
8.	Sunflower seeds	38	±	21
9.	Walnuts	12	±	14

## Data Availability

Data presented in this article are available upon request from the corresponding authors.

## Supplementary Materials

The following supporting information can be downloaded at: https://www.mdpi.com/article/10.3390/molecules28052395/s1, Tables S1: Analytical eco-scale of the DES-RP-DLLME-HPLC method for tryptophan in oils. Table S2: GAPI of the DES-RP-DLLME-HPLC method for tryptophan in oils. Table S3: The content of tryptophan in nut vegetables from the following stores: A. JEZGRO (address: Ruzveltova 24, Belgrade); B. BIO ŠPAJZ (address: Bulevar Kralja Aleksandra 297, Belgrade); C. ZDRAVAC (address: Čumićevo sokače, Lokal 45, Belgrade); D. BIO MARKET (address: Svetogorska 18, Belgrade); E. DREN (address: Bulevar Despota Stefana 90, Belgrade); F. BIO SHOP (address: Braće Jerković 116, Belgrade); G. (address: EFEDRA, Prvomajska 8k, Zemun).
