# Supplementary material for: Deep Eutectic Solvent Based Reversed-Phase Dispersive Liquid–Liquid Microextraction and High-Performance Liquid Chromatography for the Determination of Free Tryptophan in Cold-Pressed Oils
Source: Molecules. 2023 Mar 5;28(5):2395. doi: 10.3390/molecules28052395 (PMC10005200; doi:10.3390/molecules28052395)
Supplement: Supplementary file 1 [file molecules-28-02395-s001.zip › molecules-2152485-supplementary.pdf]

SUPPLEMENTARY MATERIALS

**Supplementary Table S1.** Analytical eco-scale of the DES-RP-DLLME-HPLC method for tryptophan in oils

| Reagents | | |
|---|---|---|
| Amount | n-hexane <10 mL (g) | 8 |
| | DES amount <10 mL (g) | 1 |
| Hazard (physical, environmental, health) | none | 0 |
| Instruments | | |
| Energy | HPLC instrument < 1.5 kWh per sample | 1 |
| | Vortex, Ultrasonic bath, Centrifuge, and Water bath < 1.5 kWh per sample | 1 |
| Occupational hazard | Analytical process | 0 |
| Waste | 1–10 mL (g) | 3 |
| | Total penalty points | 14 |
| | Eco-scale score | 86 |

**Supplementary Table S2.** GAPI of the DES-RP-DLLME-HPLC method for tryptophan in oils

| | |
|---|---|
| 1. Collection: | off-line |
| 2. Preservation: | None |
| 3. Transport: | None |
| 4. Storage: | Under normal conditio |
| 5. Type of method: | Simple procedures |
| 6. Scale of extraction: | Micro-extraction |
| 7. Solvents/reagents used: | Green solvents/reagent |
| 8. Additional treatments: | Simple treatments |
| **Reagents and solvents** | |
| 9. Amount: | < 10 mL (< 10 g) |
| 10. Health hazard: | Slightly toxic, slight irri |
| 11. Safety hazard: | Highest NFPA flammal |
| **instrumentation** | |
| 12. Energy: | <= 1.5 kWh per sample |
| 13. Occupational hazard: | Hermetic sealing of the |
| 14. Waste: | 1-10 mL (1-10 g) |
| 15. Waste treatment: | No treatment |
| **Method type** | |
| Type of analysis: | Qualitative and quantit |

**Supplementary Table S3.** The content of tryptophan in nut vegetables from the following stores: A. JEZGRO (address: Ruzveltova 24, Belgrade); B. BIO ŠPAJZ (address: Bulevar Kralja Aleksandra 297, Belgrade); C. ZDRAVAC (address: Čumićevo sokače, Lokal 45, Belgrade); D. BIO MARKET (address: Svetogorska 18, Belgrade); E. DREN (address: Bulevar Despota Stefana 90, Belgrade); F. BIO SHOP (address: Braće Jerković 116, Belgrade); G. (address: EFEDRA, Prvomajska 8k, Zemun).

1. Almond

| Samples | Replicates | | | Store | $Mean_i$ | $SD_i$ |
|---|---|---|---|---|---|---|
| | 1 | 2 | 3 | | | |
| 1 | 15.8 | 16.9 | 14.9 | A | 15.9 | 1.04 |
| 2 | 16.7 | 16.6 | 17.1 | B | 16.8 | 0.30 |
| 3 | 14.5 | 16.7 | 15.2 | C | 15.5 | 1.08 |
| 4 | 14.9 | 14.8 | 16.3 | E | 15.3 | 0.85 |
| 5 | 15.8 | 15.1 | 17.1 | F | 16.0 | 0.98 |
| | | | | | Mean | %MAXrange |
| | | | | | 15.9 | 16.4 |

2. Brazilian nut

| Samples | Replicates | | | Store | $Mean_i$ | $SD_i$ |
|---|---|---|---|---|---|---|
| | 1 | 2 | 3 | | | |
| 1 | 14.3 | 12.8 | 14.5 | A | 13.8 | 0.92 |
| 2 | 15.3 | 14.2 | 13.5 | C | 14.3 | 0.90 |
| 3 | 14.3 | 14.9 | 13.9 | D | 14.4 | 0.50 |
| 4 | 12.9 | 14.8 | 13.5 | E | 13.7 | 0.99 |
| 5 | 15.0 | 13.8 | 13.5 | G | 14.1 | 0.78 |
| | | | | | Mean | %MAXrange |
| | | | | | 14.1 | 18.1 |

3. Cashew

| Samples | Replicates | | | Store | $Mean_i$ | $SD_i$ |
|---|---|---|---|---|---|---|
| | 1 | 2 | 3 | | | |
| 1 | 10.6 | 11.3 | 11.5 | B | 11.2 | 0.48 |
| 2 | 10.1 | 10.2 | 10.9 | D | 10.4 | 0.42 |
| 3 | 10.8 | 10.1 | 11.8 | E | 10.9 | 0.82 |
| 4 | 10.1 | 11.5 | 10.4 | F | 10.6 | 0.72 |
| 5 | 11.3 | 10.6 | 11.4 | G | 11.1 | 0.42 |
| | | | | | Mean | %MAXrange |
| | | | | | 10.8 | 15.3 |

4. Hazelnut

| Samples | Replicates | | | Store | $Mean_i$ | $SD_i$ |
|---|---|---|---|---|---|---|
| | 1 | 2 | 3 | | | |
| 1 | 16.3 | 17.0 | 16.6 | A | 16.6 | 0.35 |
| 2 | 17.6 | 18.4 | 17.2 | B | 17.7 | 0.63 |
| 3 | 18.2 | 16.0 | 16.7 | C | 17.0 | 1.13 |
| 4 | 18.4 | 17.6 | 16.5 | F | 17.5 | 0.97 |
| 5 | 18.1 | 17.9 | 17.4 | G | 17.8 | 0.35 |
| | | | | | Mean | %MAXrange |
| | | | | | 17.3 | 13.9 |

5. Peanuts

| Samples | Replicates | | | Store | $Mean_i$ | $SD_i$ |
|---|---|---|---|---|---|---|
| | 1 | 2 | 3 | | | |
| 1 | 17.5 | 19.0 | 18.8 | B | 18.4 | 0.84 |

| | | | | | | |
|---|---|---|---|---|---|---|
| 2 | 17.4 | 17.8 | 18.6 | C | 18.0 | 0.62 |
| 3 | 17.7 | 16.7 | 16.8 | E | 17.1 | 0.56 |
| 4 | 17.3 | 16.8 | 16.9 | F | 17.0 | 0.26 |
| 5 | 17.8 | 19.1 | 16.8 | G | 17.9 | 1.12 |
| | | | | | Mean | %MAXrange |
| | | | | | 17.7 | 13.1 |

6. Pumpkin seeds

| Samples | Replicates | | | Store | $Mean_i$ | $SD_i$ |
|---|---|---|---|---|---|---|
| | 1 | 2 | 3 | | | |
| 1 | 35.0 | 29.3 | 34.4 | A | 32.9 | 3.13 |
| 2 | 30.7 | 28.1 | 36.4 | B | 31.7 | 4.22 |
| 3 | 33.3 | 28.5 | 30.7 | D | 30.8 | 2.40 |
| 4 | 35.7 | 32.5 | 28.4 | E | 32.2 | 3.64 |
| 5 | 29.2 | 32.3 | 35.3 | F | 32.3 | 3.06 |
| | | | | | Mean | %MAXrange |
| | | | | | 32.0 | 25.8 |

7. Sesame seeds

| Samples | Replicates | | | Store | $Mean_i$ | $SD_i$ |
|---|---|---|---|---|---|---|
| | 1 | 2 | 3 | | | |
| 1 | 35.8 | 35.3 | 31.5 | B | 34.2 | 2.33 |
| 2 | 30.7 | 31.8 | 36.8 | C | 33.1 | 3.25 |
| 3 | 31.6 | 36.9 | 35.3 | D | 34.6 | 2.72 |
| 4 | 31.9 | 29.2 | 28.1 | F | 29.7 | 1.97 |
| 5 | 35.3 | 33.0 | 31.4 | G | 33.2 | 1.99 |
| | | | | | Mean | %MAXrange |
| | | | | | 33.0 | 26.7 |

8. Sunflower seeds

| Samples | Replicates | | | Store | $Mean_i$ | $SD_i$ |
|---|---|---|---|---|---|---|
| | 1 | 2 | 3 | | | |
| 1 | 40.7 | 34.3 | 35.0 | A | 36.7 | 3.51 |
| 2 | 35.4 | 40.7 | 37.3 | B | 37.8 | 2.68 |
| 3 | 39.7 | 36.9 | 41.2 | C | 39.3 | 2.17 |
| 4 | 34.9 | 37.1 | 36.3 | D | 36.1 | 1.12 |
| 5 | 36.6 | 36.7 | 42.2 | G | 38.5 | 3.19 |
| | | | | | Mean | %MAXrange |
| | | | | | 37.7 | 20.9 |

9. Walnut

| Samples | Replicates | | | Store | $Mean_i$ | $SD_i$ |
|---|---|---|---|---|---|---|
| | 1 | 2 | 3 | | | |
| 1 | 10.8 | 11.5 | 11.5 | A | 11.3 | 0.40 |
| 2 | 12.5 | 11.8 | 12.0 | C | 12.1 | 0.37 |
| 3 | 12.2 | 11.2 | 12.4 | E | 11.9 | 0.68 |
| 4 | 12.4 | 12.3 | 12.2 | F | 12.3 | 0.14 |
| 5 | 12.0 | 12.2 | 12.0 | G | 12.0 | 0.13 |
| | | | | | Mean | %MAXrange |
| | | | | | 11.9 | 14.3 |

$Mean_i$ – The mean value for triplicates;
$SD_i$ – The standard deviation for triplicates;
%MAXrange – Range between the minimum and maximum individual results.
